# Comparative Proteomic and Transcriptomic Analysis of the Impact of Androgen Stimulation and Darolutamide Inhibition

**DOI:** 10.3390/cancers15010002

**Published:** 2022-12-20

**Authors:** Ekaterina Nevedomskaya, Tatsuo Sugawara, Simon J. Baumgart, Ralf Lesche, Hannes Hahne, Dominik Mumberg, Bernard Haendler

**Affiliations:** 1 Bayer AG, Pharmaceuticals, Research & Early Development Oncology, 13353 Berlin, Germany; 2 OmicScouts GmbH, 85354 Freising, Germany

**Keywords:** prostate cancer, androgen regulation, proteomics, transcriptomics

## Abstract

**Simple Summary:**

Mass spectrometry-based proteomic analysis of the VCaP prostate cancer cells treated with androgen and the androgen receptor (AR) signaling inhibitor darolutamide revealed a generally good agreement with transcriptomic responses. In some cases, however, the magnitude of changes induced in gene expression levels and in the corresponding protein levels differed, indicating post-transcriptional regulation mechanisms.

**Abstract:**

Several inhibitors of androgen receptor (AR) function are approved for prostate cancer treatment, and their impact on gene transcription has been described. However, the ensuing effects at the protein level are far less well understood. We focused on the AR signaling inhibitor darolutamide and confirmed its strong AR binding and antagonistic activity using the high throughput cellular thermal shift assay (CETSA HT). Then, we generated comprehensive, quantitative proteomic data from the androgen-sensitive prostate cancer cell line VCaP and compared them to transcriptomic data. Following treatment with the synthetic androgen R1881 and darolutamide, global mass spectrometry-based proteomics and label-free quantification were performed. We found a generally good agreement between proteomic and transcriptomic data upon androgen stimulation and darolutamide inhibition. Similar effects were found both for the detected expressed genes and their protein products as well as for the corresponding biological programs. However, in a few instances there was a discrepancy in the magnitude of changes induced on gene expression levels compared to the corresponding protein levels, indicating post-transcriptional regulation of protein abundance. Chromatin immunoprecipitation DNA sequencing (ChIP-seq) and Hi-C chromatin immunoprecipitation (HiChIP) revealed the presence of androgen-activated AR-binding regions and long-distance AR-mediated loops at these genes.

## 1. Introduction

Prostate cancer is a leading cancer type in men worldwide, and about 268,490 new cases are expected in the United States in 2022 [1]. Initial treatments include prostatectomy, local irradiation, and androgen deprivation therapy (ADT) by chemical or surgical castration [2,3]. The observation that androgen production by the adrenal glands or by the tumor itself is still able to fuel proliferation led to the development and approval of androgen receptor (AR)-targeted therapies with pharmaceutical drugs such as enzalutamide, apalutamide or darolutamide, which turn off AR function, or abiraterone, which inhibits the CYP17,20 lyase involved in androgen synthesis [2,3,4,5]. Unfortunately, therapy resistance ultimately occurs, and late-stage tumors are treated with the taxanes docetaxel and cabazitaxel, or with radium-223, in the case of symptomatic bone metastases and no known visceral metastases [6]. A deeper investigation of the molecular mechanisms involved in response and later in resistance to AR inhibitors will be essential to further understand how currently approved drugs precisely work and to determine which liabilities to address in future targeted therapies [7].

We focused on mechanisms proximal to the AR, as this nuclear hormone receptor represents the essential driver at different stages of prostate cancer. The AR controls the expression of numerous genes essential for the normal physiological role of androgens, mainly dihydrotestosterone (DHT). This is, however, altered in prostate cancer, where the AR elicits an aberrant gene expression program due to gain or loss of binding sites [8,9,10] and to reactivation of developmental epigenetic processes [11], ultimately leading to uncontrolled tumor proliferation. Numerous studies report that following androgen activation the AR sheds its chaperone proteins, moves into the cell nucleus and binds to specific DNA response elements. There, it interacts with a number of cofactors, including epigenetic regulators and pioneer factors, to form signaling complexes at promoter, enhancer and super-enhancer regions that will modulate downstream gene transcription [12,13]. AR antagonists prevent binding of the physiological androgens and reduce nuclear translocation and genome-wide binding of the AR [4,9,12,13]. Structural dynamics approaches show that agonists and antagonists induce distinct conformational changes in the AR ligand-binding domain and underline the essential role of helix 12 in cofactor binding [14]. Indeed, altered recruitment of cofactors following antagonist binding has been reported [15,16]. This ultimately leads to dramatic changes in the genome-wide binding of the AR and of factors essential for transcriptional activity such as FOXA1, as well as in histone activation marks [13,17]. The first studies on the downstream impact of antagonist binding on global gene expression in prostate cancer cells have recently been published for enzalutamide and darolutamide [13,18,19] however, there is limited information on the downstream effect on protein levels. This is particularly relevant for the AR signaling pathway, as it impacts the translation initiation process so that an additional level of regulation may be expected [20,21]. Recent comparative studies performed in benign prostate hyperplasia, primary prostate cancer and castration-resistant prostate cancer (CRPC) indicate that changes in the proteome are only partially predicted by gene copy number or RNA expression levels, which may be explained partly by the heterogeneity of the material tested but also suggests that regulatory mechanisms may take place at the post-transcriptional and translation levels [22,23].

Here we determined the impact of darolutamide, an AR antagonist with significant clinical efficacy in non-metastatic CRPC and metastatic hormone-sensitive prostate cancer [24,25] on the prostate cancer proteome. We first determined the direct binding between darolutamide and the AR in living prostate cancer cells in a label-free context using the cellular high throughput thermal shift assay (CETSA HT). We then generated comprehensive proteomic profiles of prostate cancer cells treated with androgen and darolutamide and compared them with transcriptomic profiles. We found a generally high concordance between proteomic and transcriptomic data, both on the level of detected expressed genes and their protein products, as well as in terms of the corresponding biological programs. However, there were cases where protein and gene abundance levels were not regulated in parallel, suggesting an additional post-transcriptional regulation step to occur in several instances.

## 2. Materials and Methods

### 2.1. Compounds

Darolutamide was synthesized at Orion Corporation (Espoo, Finland). DHT and the synthetic androgen R1881 are available from Sigma-Aldrich (Saint Louis, MO, USA). Details on R1881 synthesis have been published [26].

### 2.2. Origin and Authentication of Cell Lines

The CWR22Pc-R1-AD1 cell line is derived from the CWR22 xenograft, which originates from the University of Minnesota and contains a single copy of the AR gene with the mutation H875Y [27]. The VCaP (CRL-2876^TM^) prostate cancer cell line was purchased from the ATCC (American Type Culture Collection, Manassas, VA, USA). It has AR wild-type gene amplification and also expresses the splice variant AR-V7. DNA fingerprinting was performed at the DSMZ (Deutsche Sammlung von Mikroorganismen und Zellkulturen, Braunschweig, Germany) for authentication. Mycoplasma contamination testing was carried out using MycoAlert (Lonza, Cologne, Germany).

### 2.3. Preparation of Samples for Further Analyses

#### 2.3.1. CETSA Samples

CWR22Pc-R1-AD1 cells were cultured in RPMI1640 supplemented with 10% fetal bovine serum, L-glutamine and penicillin/streptomycin. Prior to treatment, the cells were washed with TrypLE (12563-029, ThermoFisher, Waltham, MA, USA), harvested, washed twice in Hank’s balanced salt solution (HBSS) and resuspended to the desired cell density in HBSS or in complete medium.

#### 2.3.2. Transcriptomic Samples

VCaP cells were grown for 2 days in hormone-deprived medium, after which they were treated either with dimethyl sulfoxide (DMSO) control, with 1 nM synthetic androgen R1881, or with 1 nM R1881 and 2 µM darolutamide. For the VCaP cells, samples for RNA isolation and further transcriptomic analysis were taken after 8 and 22 h of treatment from 5 replicates for the R1881 and darolutamide groups, and from 8–10 replicates for the DMSO or R1881 groups. Sequencing was performed via single-end, 50 base-pair reads, with an average depth of 21 million reads per sample.

#### 2.3.3. Proteomic Samples

VCaP samples were taken at 14 and 28 h after treatment start to account for the delay between gene transcription and protein translation. This delay is very close to the one reported to lead to the best correlation between global mRNA and protein fold-changes in a dynamic profiling study [28]. Triplicate samples were processed for each treatment point. The cells were lyzed, proteins were digested with trypsin and peptides were fractionated off-line by high-pH reverse phase into 6 fractions. Single fractions were further measured for 2 h each by nanoscale liquid chromatography MS/MS analysis. The MaxQuant data processing suite (Max Planck Institute of Biochemistry, Martinsried, Germany, Version 1.6.2.6), in combination with the proprietary data analysis workflow of OmicScouts (Freising, Germany), was used for peptide and protein identification and for label-free quantification. On average, 8420 proteins were identified, and 7427 proteins showing signals in three replicates of at least one condition were selected. MaxQuant label-free quantitation normalization was applied [29]. For comparison of average abundance levels, proteomic data were further normalized to the number of theoretically observable peptides to provide intensity-based absolute quantification (iBAQ) values [30].

#### 2.3.4. AR ChIP-Seq and HiChIP Samples

For AR ChIP-seq and HiChIP analysis, a total of 8 million VCaP cells were seeded in 15 cm plates and starved for 2 days. Treatment was with 1 nM R1881 only or with 1 nM R1881 and 2 µM darolutamide for 22 h before AR ChIP analysis [13]. Treatment was with 1 nM R1881 for 22 h before HiChIP analysis [31].

### 2.4. AR CETSA HT Assay

The AR CETSA HT assay is based on the AlphaScreen fluorescence resonance energy transfer (FRET) technology [32] and was optimized to precisely quantify AR engagement by darolutamide. A cell density of 40,000/well and the CETSA-BUF2 lysis buffer (PerkinElmer, Waltham, MA, USA) were selected, as these conditions yielded the best signal to background ratio (not shown). The immunoassay buffer AL000F (PerkinElmer) was used to dilute antibodies and alpha beads, following the manufacturer´s protocol. The monoclonal mouse anti-human antibody M356201-2 (Dako, Glostrup, Denmark) and the polyclonal rabbit anti-human antibody 06-680 (Merck Millipore, Burlington, MA, USA) were used for AR detection, as previously described [32]. AlphaLISA acceptor beads conjugated to anti-rabbit IgG antibody (AL104, PerkinElmer) were used to couple to the mouse primary antibodies at a final concentration of 10 μg/mL. AlphaLISA donor beads conjugated to anti-mouse IgG antibody (AS104D, PerkinElmer) were used to couple to the rabbit primary antibodies at a final concentration of 40 μg/mL. For comparison, AlphaLISA acceptor beads conjugated to anti-mouse IgG antibody (AL105C, PerkinElmer) and AlphaLISA donor beads conjugated to anti-rabbit IgG antibody (AS105M, PerkinElmer) were used. Signals were measured after overnight incubation using 615 nm luminescence emission and an Envision Alpha plate reader (PerkinElmer). Isothermal concentration-response curves were generated starting with 20 million CWR22Pc-R1-AD1 cells/mL per 15 µL well in a 96-well plate. Equal volumes of dose ranges of DHT and darolutamide were added before incubation at 37 °C for 1 h. The treated cells were heated at 46 °C, as established previously from melt and shift curves [32], for 3 min, cooled on ice, and then lyzed by the addition of CETSA-BUF2 lysis buffer and 30 min shaking at room temperature. Then, 5 μL of the samples was transferred in duplicate to a 384-well alpha detection plate, followed by addition of 4 μL of antibody mixture. After 1 h incubation, 4 μL of the acceptor beads (10 μg/mL final concentration) was added, followed by 1 h incubation and the subsequent addition of 4 μL of donor beads at 40 μg/mL final concentration. Alpha signals were determined after overnight incubation using an Envision Alpha Plate Reader. For data analysis, the raw signals and normalized intensities were analyzed and plotted using GraphPad Prism (GraphPad Software, La Jolla, CA, USA). The experiments were run in four biological repeats and eight technical replicates to yield biological statistically significant data, given assay noise and variability. Schild plots were generated for estimation of the Ki values. This was done using the EC_50_ values determined at each antagonist concentration (A′) normalized to the corresponding EC_50_ value in the absence of antagonist (A) as [Log10((A′/A)-1)]. Positive values were then used to predict the pA2 (i.e., the curve’s intersection with zero), from which the Ki value can be calculated as 10^−pA^.

### 2.5. Transcriptomic Data Generation and Analysis

Transcriptomic analysis of VCaP cells treated with R1881 and darolutamide has previously been described [13]. FASTQ reads were mapped via STAR aligner to the human genome GRCh38 and quantified with RSEM (University of Wisconsin-Madison, Madison, WI, USA, v1.3.0). Only protein-coding genes with at least 1 transcript per million (TPM) in at least 3 samples were used for further analysis. Differentially expressed genes were identified with DESeq2 [33].

### 2.6. Proteomic Data Generation and Analysis

Over 6000 proteins were quantified in each condition with label-free quantification (LFQ). Only proteins with signals in all replicates of at least one condition were used further for the analysis. Missing values were substituted with a random value drawn from a normal distribution corresponding to the lower end of protein abundance values (lower 10%). Differential protein expression was analyzed using methodology from the limma expression analysis package [34] with fitting of a linear model and empirical Bayes statistics for differential expression analysis. Data are available from the ProteomeXchange Consortium via the PRIDE partner repository under PXD036962 [35].

### 2.7. Statistical Analyses of Proteomic and Transcriptomic Data

The joint analyses of proteomic and transcriptomic data were performed with the statistical program language R (R Foundation for Statistical Computing, Vienna, Austria, version 4.0.4.) using stats and base packages. The linear model was fitted to protein and gene expression log-fold changes in cells treated with androgen and darolutamide, compared to only androgen treatment. Outliers, that is, proteins with expression fold-changes not consistent with gene expression fold-changes, were defined based on standardized residuals larger than 3.

### 2.8. ELISA Methods

VCaP cells grown in charcoal-stripped fetal calf serum medium were treated for 18 or 32 h with 1 nM R1881 and 2 µM darolutamide. Whole cell lysates were prepared and analyzed with the following kits: Human AMACR/P504S ELISA kit LS-F68619, LSBio (Seattle, WA, USA); Human FKBP51 ELISA kit, EH194RB, Thermo Fisher Scientific (Waltham, MA, USA); Human Hydroxymethylglutaryl-CoA Synthase, Mitochondrial (HMGCS2) ELISA kit, abx385022, Abbexa Ltd. (Cambridge, UK); Total PSA/KLK3 Sandwich ELISA kit, #14119C, Cell Signaling Technology (Danvers, MA, USA).

### 2.9. AR ChIP-Seq Analysis

Treated VCaP cells were processed as described for genome-wide determination of AR peaks using the specific antibody ab74272 (Abcam, Cambridge, UK) [13]. The experiments were performed in biological triplicate, and the libraries were prepared for sequencing on a HiSeq2500 Illumina machine followed by mapping to the human genome hg19. Details on the bioinformatics analysis have been published [13], and raw and processed data are available under GSE148358.

### 2.10. AR HiChIP Analysis

The protein-centric chromatin conformation method HiChIP was performed as previously described [31]. Crosslinking was done with 1% formaldehyde for ten minutes at room temperature, and chromatin complexes were immune-precipitated with the AR-specific antibody 06-680 (Merck Millipore, Burlington, MA, USA). Sequencing libraries were prepared with the MicroPlex Library Preparation Kit v2, following the manufacturer’s protocol (Diagenode SA, Seraing, Belgium), and sequenced by paired-end runs with 75 bp read length. Paired-end reads were mapped to the human reference genome GRCh37 and analyzed by the hichipper toolkit (http://aryeelab.org/hichipper) (accessed on 29 August 2019) on AR binding sites as anchor loops with default conditions. Visualization of the chromatin loops was performed using the diffloop R Bioconductor package (https://github.com/aryeelab/diffloop) (accessed on 29 August 2019).

## 3. Results

### 3.1. Darolutamide Is a Potent Antagonist of DHT Binding to the Human AR in the Cellular Environment

**Figure 1:**
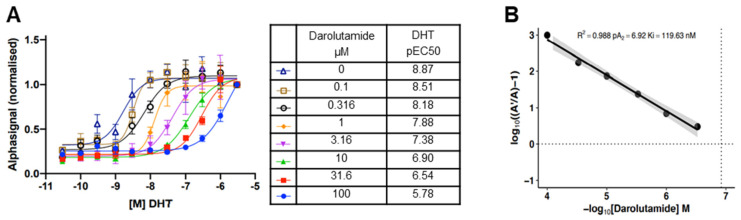
Competitive Ki screening. (**A**) CETSA EC_50_ analysis of AR in intact CWR22Pc-R1-AD1 cells treated with DHT, DHT and darolutamide, or DMSO control. Normalized AlphaLISA signals from intact cell samples heated to 46 °C after incubation with a 7-step dilution series of darolutamide (N = 4; n = 8) in the presence of serially diluted DHT. (**B**) Schild plot showing the fold change in agonist CETSA EC_50_ at the indicated darolutamide concentration, where A´ is the darolutamide concentration normalized to the corresponding EC_50_ value in the absence of darolutamide (**A**).

The CETSA HT AR assay uses the AlphaScreen FRET technology to determine agonist and antagonist binding [32]. Antibody pairs directed against the human AR were evaluated to select those giving the best specific signals, and optimization of concentration was then performed (not shown). The assay was used to determine the impact of the physiological androgen DHT and of darolutamide on the thermal stabilization of the AR in intact cells. We selected the CWR22Pc-R1-AD1 prostate cancer cell line, which expresses a single copy of the AR gene with the AR H875Y mutation [32] and is derived from the primary human prostate tumor CWR22. It recapitulates key aspects of prostate cancer, such as stimulation by androgen and inhibition by AR antagonist [36]. Importantly, the antagonistic activity of darolutamide and other antiandrogens for this AR mutant is comparable to that measured for wild-type AR [16]. Isothermal curves were generated at 46 °C in the presence of 1 nM to 5 µM DHT with a range of darolutamide concentrations from 0 to 100 µM. The normalized alpha signals and calculated pEC50 values for each darolutamide concentration are shown (Figure 1A). DHT treatment led to thermal stabilization of the AR, and this was reverted in a concentration-dependent manner by darolutamide, demonstrating competitive antagonism of DHT interaction with the AR. The measured EC_50_ values were plotted against the corresponding darolutamide concentrations as a Schild plot analysis. A linear regression was applied, and a Ki value of 119.5 nM determined (confidence interval 49.1–291.6 nM) for darolutamide, indicating a strong interaction with the AR in the cellular context (Figure 1B).

### 3.2. Most Expressed Genes Are Also Detected on Protein Level

**Figure 2:**
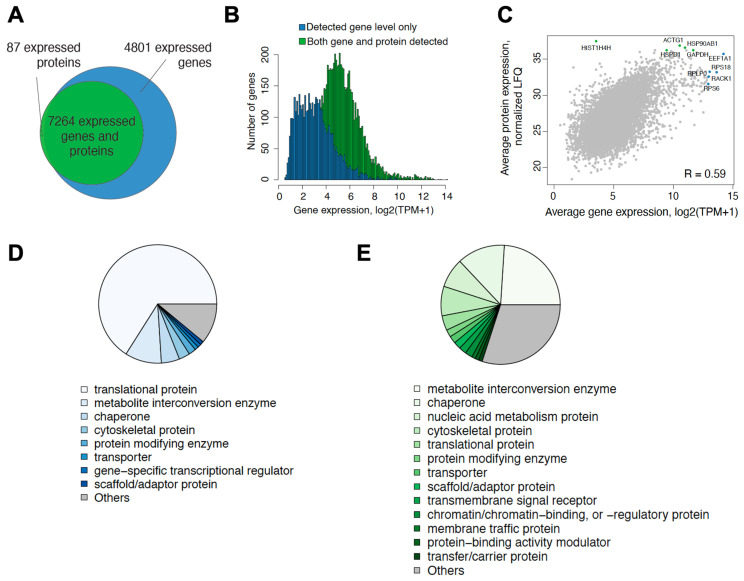
Comparison of expressed protein-coding genes and proteins detected in VCaP cells across all treatment conditions. For proteomic data analysis, proteins with signals in three replicates of at least one condition (N = 7427) were selected. (**A**) Euler diagram showing the overlap between the expressed protein-coding genes and corresponding proteins at both time points. (**B**) Histogram showing average expression (TPM) of genes detected only in RNA-seq (blue) or in both RNA-seq and proteomic data (green). (**C**) Scatter plot of average mRNA and protein expression. The top five most abundant genes and proteins are marked in blue and green, respectively. (**D**) Pie chart showing the functional classification of the most abundant genes (top 100 based on average expression across all treatment conditions). (**E**) Pie chart showing the functional classification of the most abundant proteins (top 100 based on average expression across all treatment conditions).

Transcriptomic and proteomic data were generated from VCaP cells, which harbor AR wild-type and are strongly stimulated by androgen and inhibited by antiandrogens [16]. VCaP cells were treated with DMSO only, with the androgen R1881 or with R1881 plus darolutamide, at two different time points for each of the omics data generated (8 and 22 h for transcriptomics, and 14 and 28 h for proteomics to account for the delay in protein synthesis relative to transcription) (Figure S1). In order to focus on the most robust data, we analyzed only protein-coding genes that were expressed in RNA-seq data with at least one transcript-per-million (TPM) in at least three samples (N = 12,065). Concerning the proteomic analysis, the biological and technical reproducibility of the dataset was excellent, thus warranting further bioinformatic and statistical analysis and data interpretation. Proteins with signals in three replicates of at least one condition (N = 7427) were selected. A high overlap between the detected genes and proteins was observed. For almost 60% of the genes seen expressed following RNA-seq analysis, the corresponding protein was also detected by proteomics (Figure 2A). Generally, the genes that were identified only by RNA-seq and not by proteomics were expressed at lower levels compared to the genes that were detected in both transcriptomic and proteomic experiments (Figure 2B). Average abundance levels of genes and proteins was positively correlated (R = 0.65), indicating general similarity in expression levels of transcripts and proteins (Figure 2C). We determined the categories of the most abundantly expressed genes and proteins based on average expression across all conditions and found that the majority were involved in processes linked to mRNA translation or to metabolite interconversion, as well as to protein folding or assembly (Figure 2D,E).

### 3.3. Androgen Stimulation and Darolutamide Inhibition Induce Largely Concordant Changes at the Gene and Protein Levels

**Figure 3:**
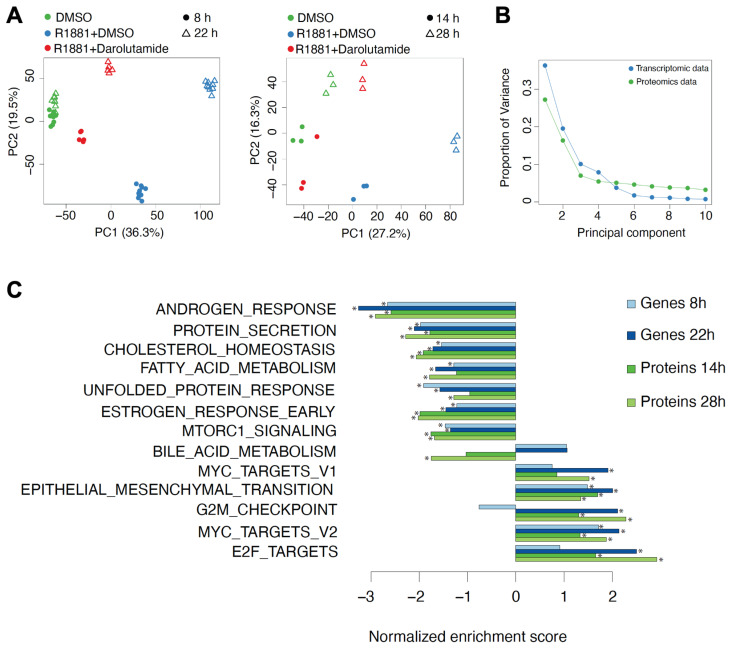
(**A**) Principal component analysis of transcriptomic and proteomic data generated from VCaP cells treated with DMSO, R1881 or R1881 and darolutamide for the indicated time. The percent of explained variance is indicated on the axes. (**B**) Proportion of variance explained by the first ten principal components in proteomic and transcriptomic data. (**C**) Top hallmark gene sets enriched among mRNAs and proteins that are down- or up-regulated at least twofold upon darolutamide and R1881 treatment, compared to R1881 treatment only. Stars (*) indicate FDR < 0.25.

Principal component analysis (PCA) demonstrated a clear separation of gene and protein expression according to treatments and short or long time points (Figure 3A). Along principal component 1, R1881 plus darolutamide samples were closer to DMSO than R1881-treated samples were, indicating the reversal of the stimulatory effect of androgen by darolutamide. The proportion of variance explained by the first three principal components was lower for proteomic than for transcriptomic data, indicating additional, potentially technical sources of variation in proteomic data (Figure 3B). We then focused on genes and proteins differentially expressed in cells treated by the androgen and darolutamide combination, compared to androgen treatment alone. Remarkable effects on known androgen-regulated genes and other androgen-dependent pathways, such as cholesterol homeostasis, fatty acid metabolism, unfolded protein response and cell cycle, were observed upon R1881 plus darolutamide application (Figure 3C). Scatter plot analysis showed a strong negative correlation between changes induced by R1881 compared to R1881 plus darolutamide, both in RNA (Figure S2A) and protein (Figure S2B) levels, demonstrating that darolutamide potently reverted expression changes induced by R1881. While hundreds of genes were found up- or down-regulated two-fold and more on the transcriptional level following treatment with R1881 and darolutamide, compared to R1881 alone, the magnitude of change was lower in the protein levels, with fewer proteins passing the two-fold threshold (Table S1).

**Figure 4:**
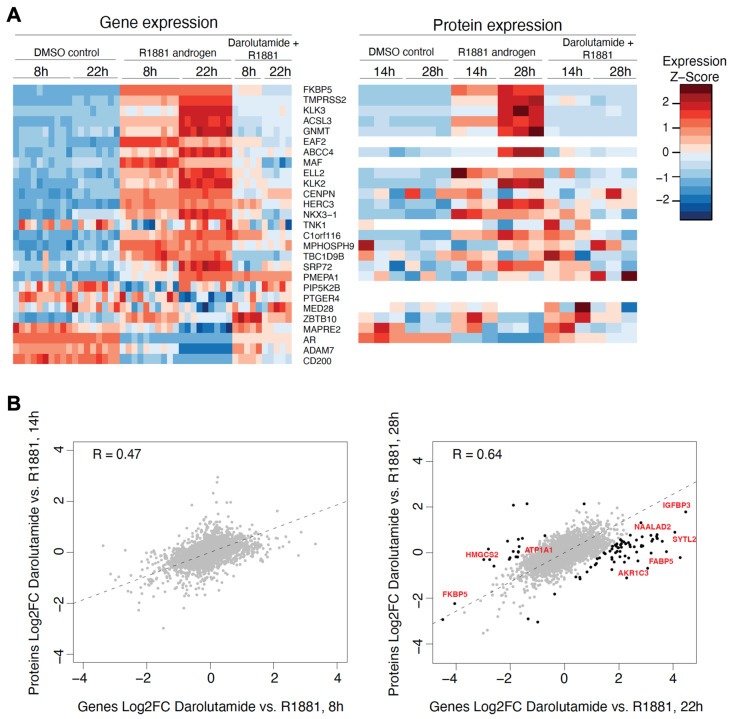
(**A**) Heatmap plots of mRNA (**left side**) and protein (**right side**) levels of AR activity signature genes following treatment of VCaP cells with DMSO, R1881, or R1881 and darolutamide for the indicated times. (**B**) Scatterplots of Log2 fold changes in mRNA and protein abundance upon R1881 and darolutamide treatment compared to R1881 treatment alone. Gene expression after 8 h and protein level after 14 h of treatment was determined as early time point (**left side**). Gene expression after 22 h and protein level after 28 h of treatment was determined as late time point (**right side**). Genes with differential magnitude of response on the mRNA and protein levels are indicated in black, a few selected androgen targets are highlighted.

For an in-depth comparison of gene expression and protein level results, we initially focused on genes from the AR activity signature [37]. In general, the regulation of gene expression by androgen and darolutamide at the transcriptional level was paralleled by regulation at the protein level, as revealed by heatmaps (Figure 4A). Expression of known AR targets, such as FK506-binding protein 5 (FKBP5), transmembrane serine protease 2 (TMPRSS2), kallikrein 3 (KLK3), acyl-CoA synthetase long chain family member 3 (ACSL3) and several other genes, was induced by androgen in a time-dependent manner both in RNA and protein levels, with expression levels being higher at a later timepoint compared to an earlier timepoint. This was strongly reversed by additional darolutamide treatment. A genome-wide analysis showed a compelling positive correlation between fold changes on RNA and protein levels upon R1881 and darolutamide treatment at the early and late time point (Figure 4B), indicating that mRNA levels are the major determinant of protein levels. Generally, the magnitude of effect was greater in RNA than in the protein level, and the correlation was higher at the later time point, probably due to time delay between transcription and translation.

### 3.4. A Discordant Impact of Darolutamide on Gene and Protein Levels Is Observed 

Despite the general concordance of expression changes on RNA and protein levels, we noticed a few cases of differential behavior, mainly at the later time points (22 h for RNA-seq and 28 h for proteomics). We therefore fitted a linear regression for RNA and protein changes upon R1881 plus darolutamide treatment, compared to R1881 treatment alone. The selected outlier genes where then analyzed using the MSigDB hallmark collection of gene sets to identify overlaps. A number of enriched gene sets, including hallmarks of androgen and estrogen response, was found; however, there was a small number of genes in each overlap (Table S2).

**Figure 5:**
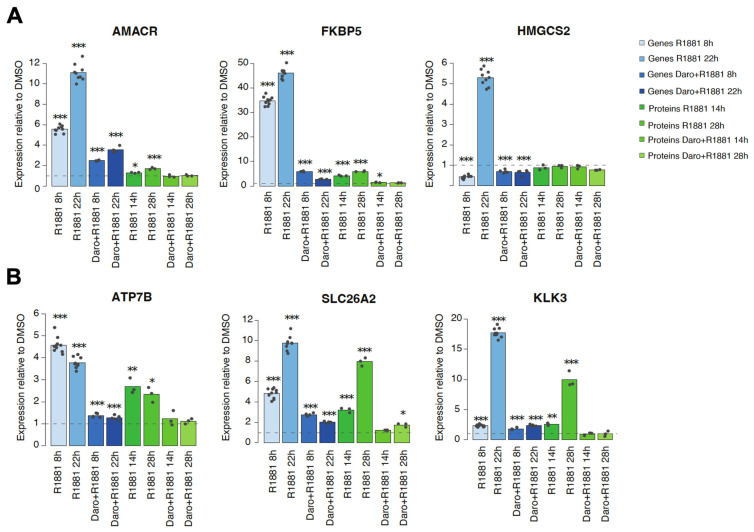
Gene and protein expression changes upon androgen, or androgen and darolutamide treatment, relative to the corresponding DMSO control which was set to 1 (grey horizontal dotted line). Blue bars indicate expression of genes measured by RNA-seq, green bars indicate levels of proteins measured by tandem mass spectrometry. (**A**) Examples of genes with discordant changes in mRNA and protein levels. (**B**) Examples of genes with similar responses at the mRNA and protein levels. Stars indicate the false discovery rate (FDR) for comparison of the indicated condition to the corresponding DMSO control: *** *p* < 0.0001, ** *p* < 0.001, * *p* < 0.01.

Examples of outlier genes constituted less than 1.5% of all proteins detected, and examples included alpha-methylacyl-CoA racemase (AMACR) and HMGCS2, for which there was little to no impact on protein levels following androgen or antagonist treatment, compared to the remarkable changes observed at the transcript levels (Figure 5A). Another striking example was FKBP5, where an increase of protein levels after androgen treatment and repression by additional darolutamide treatment was observed, but this was far less pronounced than the changes observed for the mRNA levels (Figure 5A). The results were confirmed at a later time point by ELISA analysis, indicating that the differential regulation of AMACR, FKBP5 and HMGCS2 was sustained (Figure S3). In comparison, for many other genes, including those for ATP7B, solute carrier family 26 member 2 (SLC26A2) and KLK3, there was a parallel effect on RNA and protein levels following androgen or androgen plus antagonist treatment (Figure 5B).

Examination of MA plots of protein level changes following androgen or androgen plus darolutamide treatment indicated that there was no relationship between belonging to the outlier category and the measured protein expression level (Figure S4).

Altogether, the differences in the effects on the transcription and protein expression levels observed for specific genes after androgen and antiandrogen treatment suggest that distinct kinetics of protein translation as well as differential post-transcriptional regulation of protein abundance occur in some instances and represent an additional important control mechanism.

### 3.5. AR Binding Peaks and AR Loops Are Found in Androgen-Regulated Genes

**Figure 6:**
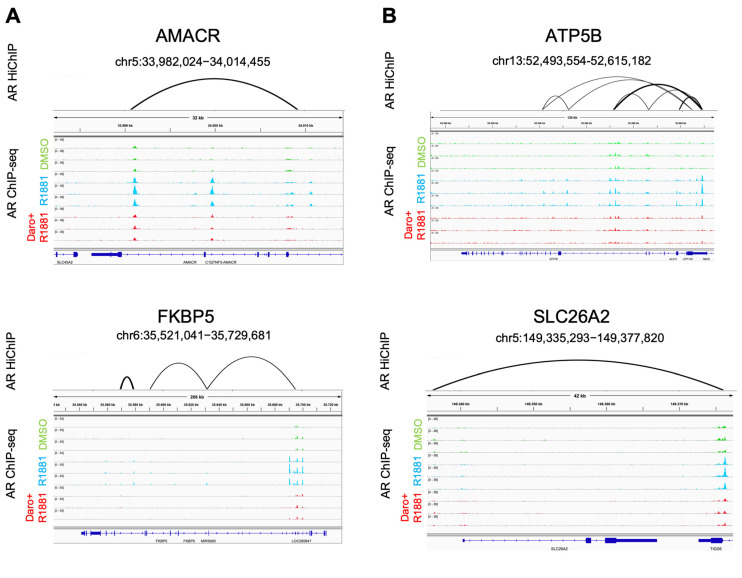

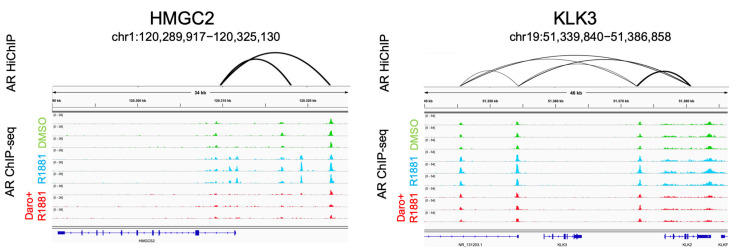
Determination of AR peaks and AR-mediated loops around selected gene regions in VCaP cells treated with DMSO, androgen, or androgen and darolutamide. (**A**) AR HiChIP and ChIP-seq results for genes with divergent regulation of RNA and protein levels. (**B**) AR HiChIP and ChIP-seq results for genes with parallel regulation of RNA and protein levels. Loop intensities are reflected by the thickness of the lines.

The outlier group is characterized by high androgen stimulation of mRNA levels but limited changes in the corresponding protein levels. In order to ascertain that the expression of these outlier genes was directly under androgen control, we examined the presence of AR peaks along the respective genomic regions. AR ChIP-seq analysis revealed in all cases several AR peaks that increased in intensity upon R1881 application and decreased again after additional darolutamide treatment (Figure 6A). We then analyzed the long-range contacts engaged in situ by the AR bound to these regions using the protein-centric HiChIP technology, and AR-associated loops were identified for the examined genes in androgen-treated cells (Figure 6A). Comparable results were obtained for genes for which a parallel androgen stimulation was observed at the mRNA and the protein levels (Figure 6B). Altogether, these findings indicate a direct binding of the AR to the genomic regions of androgen-dependent genes and the additional formation of AR-mediated long-range interactions, also in the outlier group. The reduced effects on protein level observed for the outlier group may therefore be a result of slow translation, high protein stability or combined translational and post-translational effects, which warrants further investigation.

## 4. Discussion

CETSA was used to determine the interaction of darolutamide with the AR in a native context. This method offers the advantages of both the target and test compound being label-free. In this assay, agonist binding leads to thermal stabilization, and this is reversed by a competitive antagonist [32]. The Ki value determined is the intracellular equivalent of the apparent binding affinity. Darolutamide showed a strong affinity for the AR in these live prostate cancer cells, which confirms the strong AR binding previously measured in extracts from rat ventral prostates [38] and the potent inhibitory activity determined in cell-based transactivation assays for wild-type AR and different mutants, including H875Y, which is expressed in the androgen-dependent CWR22Pc-R1-AD1 model used here [16,38]. Hopefully, the CETSA technology will be expanded further to allow measurements in co-culture systems or in organoids that better reflect the cellular microenvironment [39], so that measuring binding affinity to the AR under these more physiological conditions will also be possible.

Several papers report that proteomic data are generally more predictive for gene function than transcriptomic data [40,41]. The correlation between mRNA and protein levels is generally estimated to be at least 40% across genes, with large variations depending on the tissue or cell samples analyzed; however, technical biases may, in part, account for this [42]. A better correspondence was found in a small-scale study for genes differentially expressed under precise experimental conditions [43]. Protein levels depend on mRNA transcription rates and half-lives, as well as on protein translation rates and half-lives [44]. Highly abundant mRNA molecules usually produce more protein numbers per transcript [45]. The degree of correlation also varies among tissues, possibly due to differences in post-translational regulation mechanisms [46]. Importantly, variabilities in protein synthesis rates are critical for tissue development and homeostasis, and the underlying mechanisms are gradually being unraveled [47].

Our deep quantitative proteomic profiling of treated VCaP cells by label-free proteomic analysis allowed us to quantify 7427 proteins with signals in three replicates of at least one condition. We compared these results with transcriptomic data generated in the same cell line under similar treatment conditions. We synchronized the division of the VCaP cells by starvation and subsequent stimulation by androgen. We also introduced a time lapse between RNA and protein determination in order to account, in some measure, for the delay in translation and allow for steady-state status to be reached. We focused on intracellular proteins, as the cell secretomes reportedly show limited correlation to transcriptomes [48]. We found a generally good concordance between proteomic and transcriptomic data based on overall protein and transcript levels, and this was also reflected in terms of the corresponding biological programs. The most abundantly overall expressed genes encoded mainly translational proteins, but this was a slightly less prominent category among the most abundantly expressed proteins, which might be explained by high translation rates and also by high stability of these proteins [30]. Androgen treatment strongly induced gene expression programs related to cell proliferation and survival, both at the transcription and protein expression levels.

The stimulatory effects we observed following androgen application were potently reverted by additional darolutamide treatment, in line with the high anti-proliferative activity of this compound in androgen-dependent prostate cancer models and its efficacy in the clinic [24,25,49]. There was an altogether tight correlation for transcript and protein levels of differentially regulated genes in the tested VCaP cells. However, for about 1.5% of significantly regulated genes, only a limited correspondence was observed. There were cases where the clear up-regulation of mRNA levels by androgen was followed by only modest effects on the corresponding protein levels. A remarkable example is FKBP5, for which we measured a 40–50 fold up-regulation by androgen at the transcript level, but far less stimulation of the corresponding protein level, suggesting post-transcriptional fine-tuning to be important to control the levels of this chaperone, which plays a critical role in AR dimerization [50]. The FKBP5 gene has been described as strongly androgen-regulated in several reports, and western blot analysis shows androgen to increase its protein levels in LNCaP cells [51]; however, there are no reports discussing the impact of its down-regulation on prostate cancer models. Importantly, molecular chaperones are essential to protect the AR from degradation, which may occur following cellular stress evoked by castration or small-molecule inhibitors [52]. Another outlier is AMACR, whose expression is elevated in prostate cancer compared to non-cancerous prostate samples [53]. AMACR is essential for lipid biosynthesis, which has an important role in fueling tumor progression [54], and expression knock-down and selective inhibition lead to reduced proliferation of several prostate cancer cell lines [55]. We found a clear impact of androgen on the AMACR transcript but not on the corresponding protein levels, suggesting an intracellular mechanism controls this step under the experimental conditions we used. Importantly, previous work shows that the AMACR protein levels are not impacted by androgen in two other androgen-dependent models, namely the LAPC-4 and LNCaP cell lines [56,57], which indicates that our findings are not limited to one cell line. HMGCS2 is differentially expressed between hormone-sensitive and hormone-resistant prostate cancer samples and belongs to a regulatory network in CRPC. It is a third outlier we identified. Its expression is associated with shorter disease-free survival and biochemical recurrence-free survival after radical prostatectomy [58], suggesting a limited correlation between the AR pathway and a role of HMGCS2 in prostate cancer, in line with the uncoupled regulation of RNA vs. protein levels that we observed.

We furthermore supplemented our findings on expression regulation of the selected genes using AR ChIP-seq and AR-based HiChIP data. Both experiments pointed to a direct regulation of the selected genes by the AR, as androgen-dependent binding and looping was observed. Interestingly, there was no clear difference observed between those androgen-regulated genes for which there was a parallel shift in protein levels and those without this shift. This is in line with post-transcriptional translation regulation mechanisms or post-translational regulation of protein life cycle such as degradation, modification or subcellular localization, ultimately affecting protein levels. Additional studies are needed to determine the precise mechanisms involved.

Previously published proteomic studies analyzing the impact of AR antagonists on prostate cancer cell lines mainly focused on early, first-generation compounds and used two-dimensional gel electrophoresis, so that far fewer proteins were analyzed than in the present study [59,60]. A more recent proteomic analysis determined the impact of the second-generation AR antagonist enzalutamide by sequential window acquisition of all theoretical mass spectra (SWATH-MS), and only a limited correlation between mRNA and protein expression was reported [61], possibly linked to the limitations of the method used and the fewer proteins identified as compared to our study. We report for the first time the impact of the newly approved AR antagonist darolutamide using a very sensitive, label-free, global mass spectrometry–based approach and show a much better concordance between the regulation of protein and mRNA levels than found in previous studies with other compounds. Importantly, darolutamide is structurally different from other second-generation AR antagonists, such as enzalutamide and apalutamide. It is characterized by a flexible polar-substituted pyrazole structure, which leads to unique interactions within the AR ligand-binding pocket and to differential recruitment of cofactors compared to other AR antagonists [16], and to differences in the downstream regulated genes [62]. Proteogenomic studies performed in prostate tumors indicate a good match between proteome and transcriptome data in early prostate cancer but a much more limited correlation in late-stage disease, possibly linked to the higher heterogeneity [22,63]. An obvious difference is that tumor samples are usually analyzed in bulk, which means that a whole collection of asynchronized tumor cells, as well as stromal and immune cells, are studied at the same time, which contrasts with our analysis where a pure cell line population, additionally synchronized upon androgen stimulation following starvation, was used. Detailed, integrative proteomic and transcriptomic analyses of additional CRPC models, for example, patient-derived xenografts [64], will therefore be essential to understand the complexity of late-stage tumors and translate in vitro findings into the clinical setting.

## 5. Conclusions

In this study, we report that the integration of transcriptomic and of proteomic data from treated prostate cancer cells yield unique information on biological systems and reveal their complementarity when it comes to understanding the dynamics of how external stimuli and targeted drugs affect the cellular phenotype. These findings will potentially be strengthened by detailed protein turnover studies and subcellular localization analyses. Additional acquisition and integration of phosphoproteomic, glycoproteomic, and protein–protein interaction and metabolic perturbation data for a multi-omics analysis will lead to better characterized model systems and further increase our knowledge of the complex mechanisms involved, ultimately supporting the development of novel therapeutic treatments for prostate cancer [65].

## Data Availability

The transcriptomic data were published previously and are available under GSE148397 [13]. The mass spectrometry proteomic data were deposited to the ProteomeXchange Consortium via the PRIDE partner repository [35] with the dataset identifier PXD036962.

## Supplementary Materials

The following supporting information can be downloaded at: https://www.mdpi.com/article/10.3390/cancers15010002/s1. Figure S1: Experimental set-up for transcriptomic and proteomic analyses of VCaP cells including the treatment groups and time of collection of material. Figure S2: Scatter plot analysis showing the overall impact of darolutamide on androgen-induced gene transcription and protein levels. Figure S3: Determination of protein levels by ELISA following treatment of VCaP cells with R1881, or R1881 plus darolutamide. Figure S4: MA plots of the differences in protein expression in treated VCaP cells. Table S1: Number of genes and proteins up- or down-regulated at least twofold following darolutamide and R1881 treatment, compared to R1881 treatment alone. Table S2: Overlap of selected outlier genes and gene sets from the hallmark MSigDB collection.
