# Supplementary material for: Comparative Proteomic and Transcriptomic Analysis of the Impact of Androgen Stimulation and Darolutamide Inhibition
Source: Cancers (Basel). 2022 Dec 20;15(1):2. doi: 10.3390/cancers15010002 (PMC9817687; doi:10.3390/cancers15010002)
Supplement: Supplementary file 1 [file cancers-15-00002-s001.zip › cancers-2043510-supplementary.pdf]

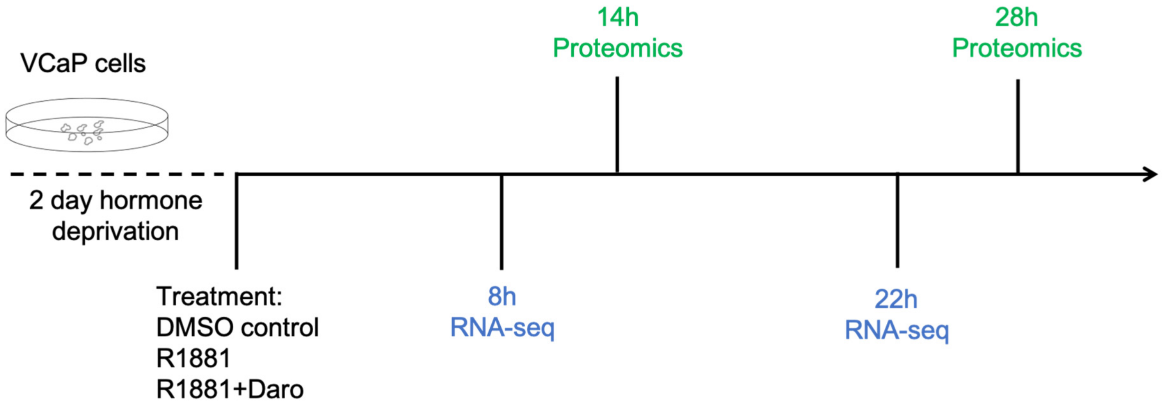

Figure S1. Experimental set-up for transcriptomic and proteomic analyses of VCaP cells including the treatment groups and time of collection of material.

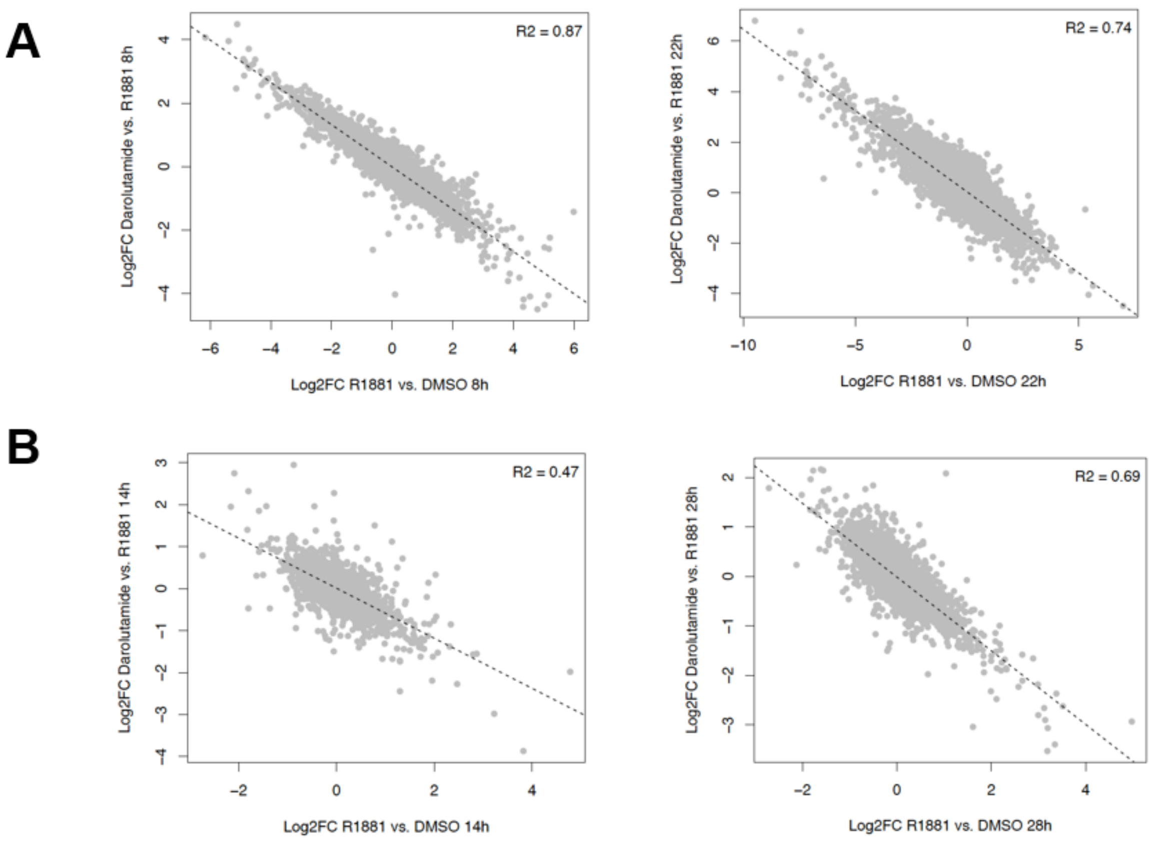

Figure S2. Scatter plot analysis showing the overall impact of darolutamide on androgen-induced gene transcription and protein levels. (**A**) Scatter plots of Log2FCs upon R1881 (on x-axes) and R1881 plus darolutamide treatments for expressed genes at 8 h (left side) and 22 h (right side). (**B**) Scatter plots of Log2FCs upon R1881 (on x-axes) and R1881 plus darolutamide treatments for expressed proteins at 14 h (left side) and 28 h (right side).

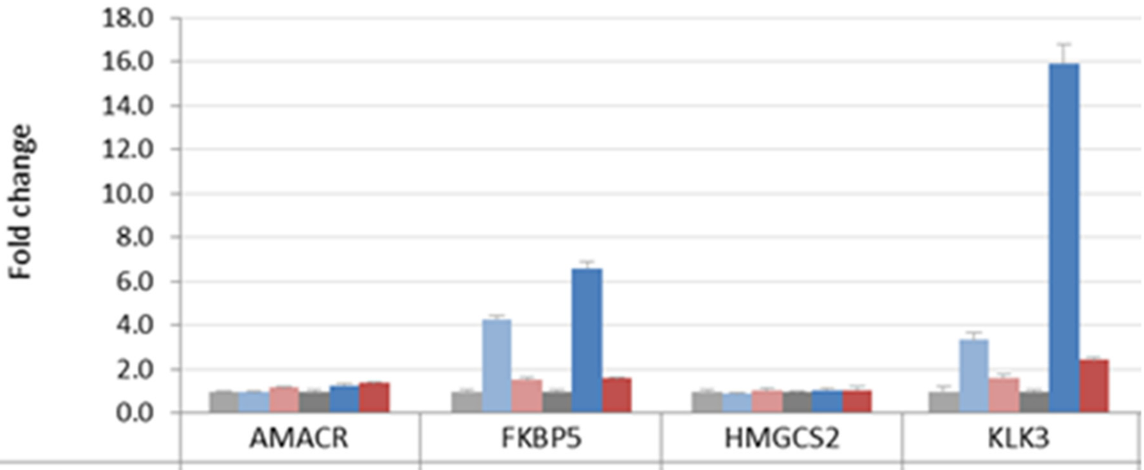

	AMACR	FKBP5	HMGCS2	KLK3
DMSO, 18h	1	1	1	1
R1881, 18h	1.0	4.3	0.9	3.3
R1881 + Daro, 18h	1.2	1.5	1.0	1.6
DMSO, 32h	1	1	1	1
R1881, 32h	1.3	6.6	1.0	15.9
R1881 + Daro, 32h	1.4	1.6	1.0	2.4

Figure S3. Determination of protein levels by ELISA following treatment of VCaP cells with R1881, or R1881 plus darolutamide. Fold changes are given for the times indicated in comparison to the corresponding DMSO level which was set to 1.

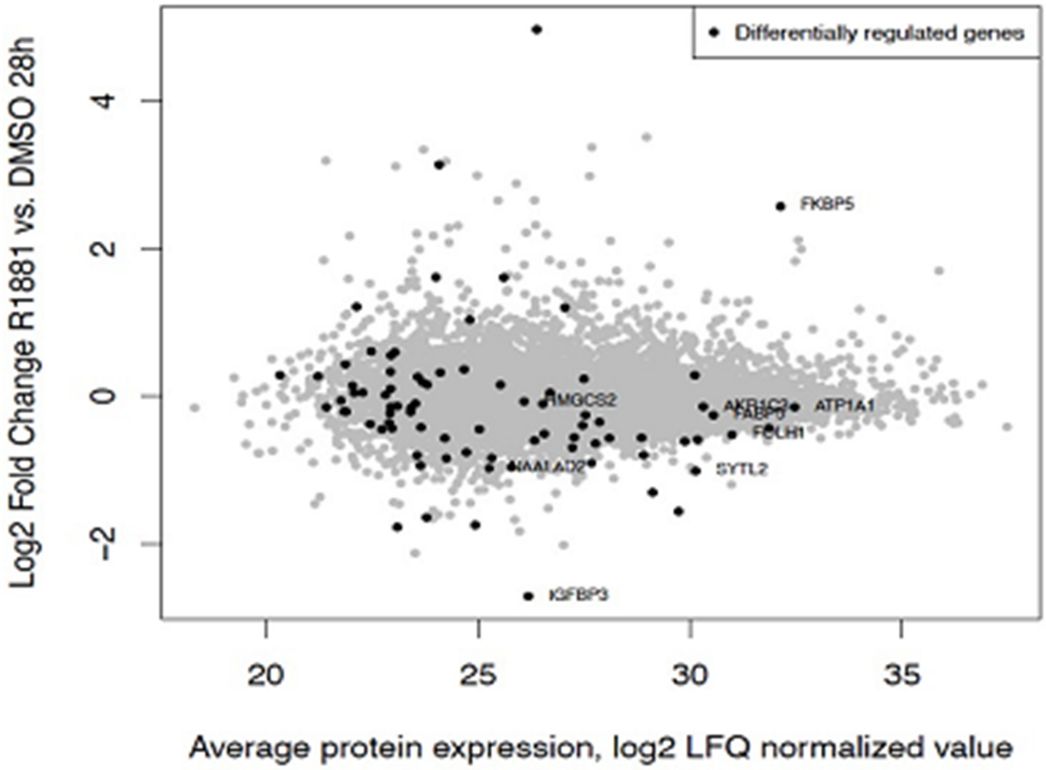

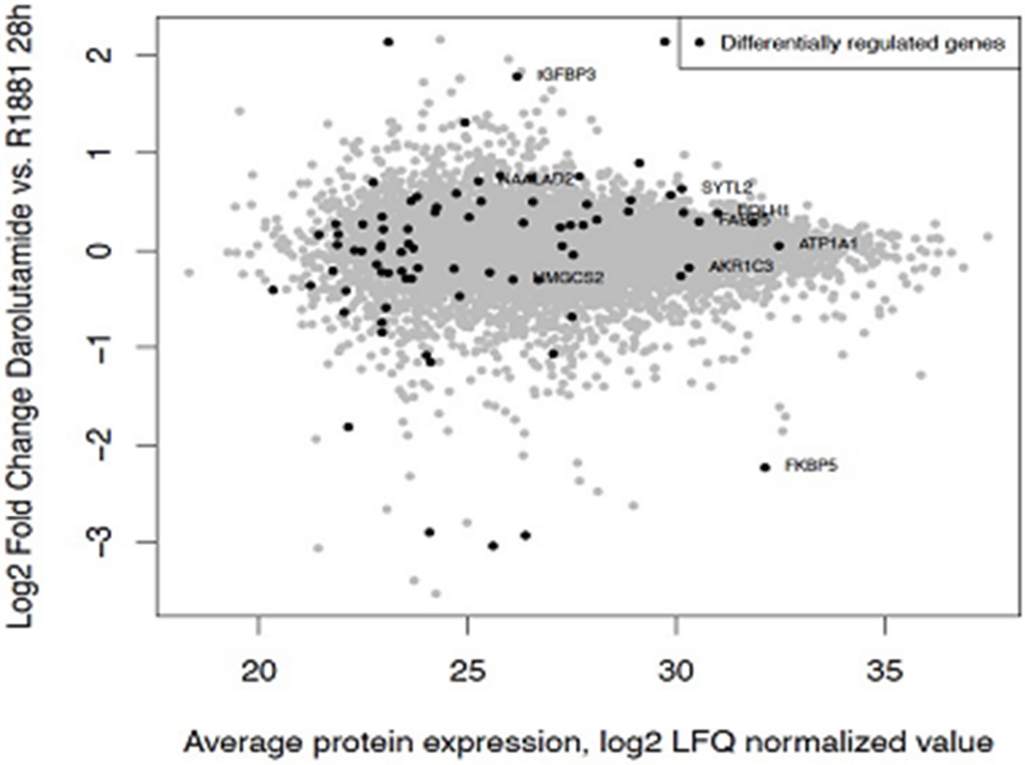

Figure S4. MA plots of the differences in protein expression in treated VCaP cells. R1881-treated cells were compared to DMSO (top) and R1881 plus darolutamide-treated cells compared to R1881-treated cells (bottom). Black dots show genes for which there is a difference in the magnitude of effect on gene and protein expression levels. Known androgen-regulated genes are marked with gene symbols.

Table S1. Number of genes and proteins up- or down-regulated at least twofold following darolutamide and R1881 treatment, compared to R1881 treatment alone.

Transcriptomics data			Proteomics data		
	**8h**	**22h**		**14h**	**28h**
**Up-regulated genes**	446	1102	**Up-regulated proteins**	22	76
**Down-regulated genes**	323	557	**Down-regulated proteins**	59	113

Table S2. Overlap of selected outlier genes and gene sets from the hallmark MSigDB collection.

Gene Set Name	# Genes in Gene Set (K)	Description	# Genes in Overlap (k)	k/K	p-value	FDR q-value
HALLMARK_ESTROGEN_RESPONSE_LATE	200	Genes defining late response to estrogen.	6	0.03	5.52E-06	2.76E-04
HALLMARK_APICAL_JUNCTION	200	Genes encoding components of apical junction complex.	4	0.02	1.01E-03	2.43E-02
HALLMARK_ANDROGEN_RESPONSE	101	Genes defining response to androgens.	3	0.0297	1.46E-03	2.43E-02
HALLMARK_SPERMATOGENESIS	135	Genes up-regulated during production of male gametes (sperm), as in spermatogenesis.	3	0.0222	3.32E-03	3.98E-02
HALLMARK_UV_RESPONSE_DN	144	Genes down-regulated in response to ultraviolet (UV) radiation.	3	0.0208	3.98E-03	3.98E-02
HALLMARK_MYC_TARGETS_V2	58	A subgroup of genes regulated by MYC - version 2 (v2).	2	0.0345	7.27E-03	4.10E-02
HALLMARK_E2F_TARGETS	200	Genes encoding cell cycle related targets of E2F transcription factors.	3	0.015	9.83E-03	4.10E-02
HALLMARK_EPITHELIAL_MESENCHYMAL_TRANSITION	200	Genes defining epithelial-mesenchymal transition, as in wound healing, fibrosis and metastasis.	3	0.015	9.83E-03	4.10E-02
HALLMARK_ESTROGEN_RESPONSE_EARLY	200	Genes defining early response to estrogen.	3	0.015	9.83E-03	4.10E-02
HALLMARK_G2M_CHECKPOINT	200	Genes involved in the G2/M checkpoint, as in progression through the cell division cycle.	3	0.015	9.83E-03	4.10E-02
